# Understanding Superior Mesenteric Artery Syndrome: Etiology, Symptoms, Diagnosis, and Management

**DOI:** 10.7759/cureus.61532

**Published:** 2024-06-02

**Authors:** Pratik S Navandhar, Raju K Shinde, Pankaj Gharde, Tushar Nagtode, Nitesh Badwaik

**Affiliations:** 1 General Surgery, Jawaharlal Nehru Medical College, Datta Meghe Institute of Higher Education and Research, Wardha, IND

**Keywords:** surgical intervention, medical treatment, duodenojejunostomy, nasogastric decompression, imaging modalities, gastrointestinal evaluation, wilkie's syndrome, sma syndrome, superior mesenteric artery syndrome

## Abstract

Superior mesenteric artery (SMA) syndrome, also known as Wilkie's syndrome, poses a diagnostic challenge due to its rarity and varied clinical manifestations. This review explores the syndrome's etiology, symptoms, diagnostic challenges, and management strategies. Symptoms range from early satiety to severe abdominal pain, often leading to malnutrition. Diagnosis involves a thorough gastrointestinal evaluation and various imaging modalities. Management includes medical interventions like nasogastric decompression and nutritional support, along with surgical interventions such as duodenojejunostomy. A thorough understanding of SMA syndrome's complexities is crucial for its timely diagnosis and effective management, especially considering its potential overlap with other gastrointestinal disorders or eating disorders. Further research is needed to enhance understanding and improve patient outcomes.

## Introduction and background

Superior mesenteric artery (SMA) syndrome is caused by extrinsic compression between the SMA and the aorta, sometimes called Wilkie's syndrome [1]. The obstruction of the duodenum results from this compression [1]. The third and last segment of the duodenum is crushed between the SMA and the abdominal aorta (AA) in this gastro-vascular condition [2]. The absence of visceral and retroperitoneal fat results in an angle between the AA and the SMA that is usually 6-25°, as opposed to the normal range of 38-56°, which can induce this uncommon and potentially fatal illness [3]. The aortomesenteric distance is also 2-8 millimeters instead of 10-20 [4].

It should be noted that there have been reports of narrow SMA angles in low BMI individuals, particularly in children who do not exhibit any SMA syndrome symptoms. Therefore, a tiny SMA angle is insufficient for diagnosis [5]. Although both disorders can be diagnosed together, they should not be confused with nutcracker syndrome, which concerns the left renal vein that is imprisoned between the SMA and the AA [6].

## Review

Search methodology

The search methodology for compiling the review article on SMA syndrome followed a systematic process in accordance with the Preferred Reporting Items for Systematic Reviews and Meta-Analyses (PRISMA) guidelines. Initially, we conducted a thorough search across databases such as PubMed, MEDLINE, Google Scholar, and Scopus, utilizing a combination of relevant keywords and Medical Subject Headings (MeSH) terms, including "Superior Mesenteric Artery Syndrome," "SMA Syndrome," "Diagnosis," "Treatment," "Aetiology," and "Symptoms," among others. Our search methodology aimed at identifying primary research articles and review papers published in English within the past decade, focusing on the aetiology, symptoms, diagnosis, treatment, and management of SMA syndrome in human subjects. We screened titles and abstracts for relevance and then performed a detailed assessment of full-text articles to determine their eligibility based on inclusion criteria. Data extraction involved retrieving pertinent information from selected studies, which was then organized thematically to correspond with the various sections of the review article. Synthesizing the extracted data enabled a comprehensive analysis of SMA syndrome, addressing its complexities and challenges.

**Figure 1:**
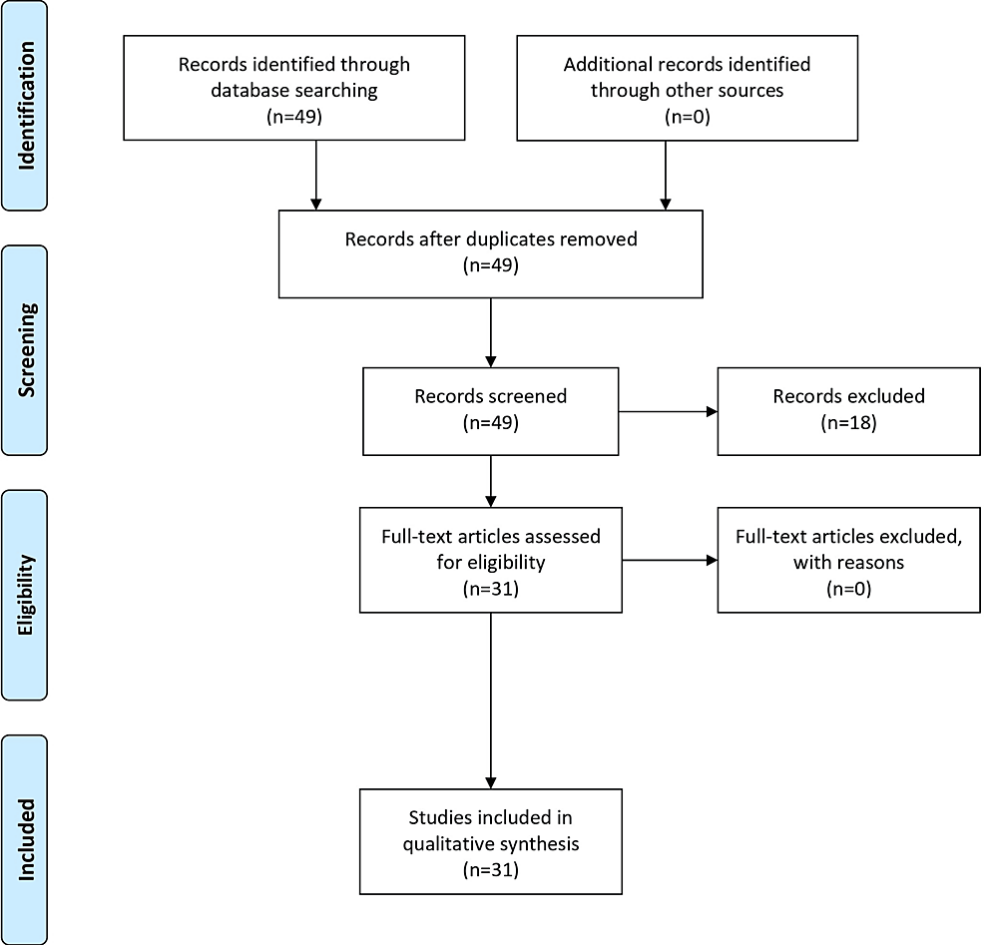
PRISMA flow diagram. PRISMA: Preferred Reporting Items for Systematic Reviews and Meta-Analyses.

Furthermore, we conducted a quality assessment to evaluate the reliability and methodological rigor of the included studies, ensuring the integrity of the review findings. By systematically following these steps, the review aimed to provide evidence-based insights into the diagnosis and management of SMA syndrome, serving as a valuable resource for clinicians, researchers, and healthcare professionals involved in tackling this rare gastrointestinal condition. The PRISMA flow diagram is presented in Figure 1.

Recognizing and managing symptoms of SMA syndrome

Early satiety, nausea, vomiting, severe "stabbing" postprandial abdominal pain (caused by compensatory reversed peristalsis and duodenal compression), burping, external hypersensitivity, stomach distention or distortion, soreness, reflux, and heartburn are among the warning signs and symptoms [7-9]. Severe malnutrition may coexist with spontaneous wasting in some SMA cases [10]. Malnutrition leads to a vicious cycle of worsening symptoms as the underlying cause progresses and duodenal constriction increases [11]. Eating anxiety is a common symptom in the chronic form of SMA syndrome. People find relief in symptoms by adopting a face-down or knee-to-chest posture [12]. The Hayes technique, which involves exerting pressure dorsally and cephalad below the umbilicus, also partially relieves the constriction [13]. Leaning to the right or standing upright can exacerbate symptoms [1].

Understanding the etiology and presentation of SMA syndrome

Usually, the duodenum is protected from the compression of the SMA by the cushioning effect of retroperitoneal fat and lymphatic tissue. Therefore, any ailment with a slight mesenteric angle and insubstantial cushion can cause SMA syndrome [14]. There are two possible presentations of SMA syndrome, namely acute and chronic [14]. People with congenital, chronic SMA syndrome usually have a long history of stomach issues with occasional exacerbations, ranging up to a lifetime based on the extent of duodenal compression [15]. Anatomical risk factors include a thin body build, an excessively high duodenal insertion at the ligament of Treitz, a notably low origin of the SMA, or intestinal malrotation along an axis created by the SMA [16]. The following conditions can easily exacerbate predisposition: impaired gastrointestinal motility, retroperitoneal tumors, appetite loss, stomach injury; an adolescent growth spurt that occurs quickly, hunger, weight loss, catabolic circumstances, abdominal wall laxity, peritoneal adhesions, malabsorption, cachexia, visceroptosis, excessive lumbar lordosis, and a history of brain damage [17]. The acute form appears rapidly following severe trauma that pushes the SMA over the duodenum forcefully, resulting in obstruction or rapid weight loss [18]. Extended periods of bed rest, surgery for scoliosis, and left nephrectomy with ileo-anal pouch surgery are among the causes [1,13,19]. Distinguishing SMA syndrome from anorexia nervosa is typically challenging [20]. For patients with SMA syndrome, regaining weight might be difficult if they also suffer from anorexia [20]. Features of acute and chronic SMA syndrome are described in Table 1.

**Table 1:** Features of acute and chronic SMA syndrome. SMA: Superior mesenteric artery.

Presentation	Characteristics
Acute SMA Syndrome	Rapid onset following severe traumas (e.g., surgery, trauma)
Chronic SMA Syndrome	Long history of stomach issues with occasional exacerbations, possibly lifelong; similar anatomical risk factors as acute
Anatomical Risk Factors	Thin body build, high duodenal insertion at ligament of Treitz, low origin of Superior Mesenteric Artery (SMA), intestinal malrotation, other anatomical anomalies
Exacerbating Factors	Impaired gastrointestinal motility, retroperitoneal tumors, appetite loss, stomach injury, rapid adolescent growth spurt, weight loss, catabolic states, abdominal wall laxity, peritoneal adhesions, malabsorption, cachexia, visceroptosis, excessive lumbar lordosis, history of brain damage
Causes	Extended bed rest, surgery for scoliosis, left nephrectomy with surgery of ileo-anal pouch
Difficulty in Diagnosis	Difficult to distinguish from anorexia nervosa
Complications	Obstruction, rapid weight loss; difficulty regaining weight, potential complications with eating disorders

Challenges in diagnosing and understanding SMA syndrome

A diagnosis of SMA syndrome is often one of exclusion and is exceedingly challenging. Therefore, SMA syndrome is only taken into consideration after patients have had a thorough evaluation of their gastrointestinal tract, which includes an upper endoscopy, and an assessment for several more commonly diagnosed conditions such as malabsorptive, ulcerative, and inflammatory intestinal disorders [21]. X-ray demonstrating duodenal dilatation, sudden constriction near the overlying SMA, and a four- to six-hour transit delay across the gastroduodenal region may be used to diagnose SMA [22]. Upper gastrointestinal series (UGI), pelvic and abdominal CT scan with oral and IV contrast, and hypotonic duodenography for equivocal cases are standard diagnostic examinations [22,23]. The identification and confirmation of SMA syndrome have been disputed despite numerous case reports, given that symptoms sometimes align differently with radiologic findings and may only sometimes improve after surgery [22,24].

Nonetheless, the continued prominence of reversed peristalsis relative to direct peristalsis has been linked to the continuation of gastrointestinal problems in certain patients, even after surgical repair [20]. Given that girls between the ages of ten and 30 are the most commonly affected, it is typical for healthcare providers to mistakenly believe that a patient's choice of emaciation is the result of SMA syndrome [25]. Early-stage SMA syndrome patients may try to adjust to the disease by reducing their food intake and opting for a lighter, easier-to-digest diet, possibly not realizing they are sick until significant harm is done to their health [23,25]. SMA syndrome diagnosis is presented in Table 2.

**Table 2:** SMA syndrome diagnosis. SMA: Superior mesenteric artery.

Feature	Description
Challenge	Diagnosis by exclusion; considered after ruling out other causes
Initial Evaluation	Upper endoscopy and assessment for more common intestinal disorders
X-ray Findings	Duodenal dilatation, constriction near SMA, delayed gastric emptying
Diagnostic Tests	Upper GI series, CT scan with contrast, hypotonic duodenography (equivocal cases)
Controversy	Symptoms may not align with imaging, surgery outcomes may vary
Persistent Symptom	Reversed peristalsis linked to ongoing issues
High-Risk Group	Females aged 10-30
Misdiagnosis	Anorexia nervosa mistaken for SMA syndrome
Patient Behavior	Early-stage patients may limit food intake, delaying diagnosis

Management strategies and surgical interventions for SMA syndrome

Acute, acquired SMA syndrome might manifest as sudden emergence during an inpatient stay after scoliosis surgery. In contrast, chronic SMA syndrome develops throughout a lifetime and advances as a result of environmental triggers, life changes, or other disorders [26]. Many modern sources state that at least 70% of cases are usually treatable with medicine, with surgery necessary for the remaining cases [23,27]. In many cases, medical treatment is tried initially. Emergency surgery may be required at the time of presentation in certain circumstances. In pediatric situations, a six-week medical therapy trial is advised [28]. The purpose of SMA syndrome medical treatment is weight gain and the resolution of underlying problems. To provide decompression of the duodenum and stomach, medical treatment may involve the insertion of a nasogastric tube, mobilization into a prone or left lateral decubitus position, replacement of fluid and electrolytes, reversal or removal of the precipitating factor, suitable nutrition, or the administration of total parenteral nutrition (TPN) via a peripherally inserted central catheter (PICC line) [1,2,29]. Metoclopramide and other pro-motility drugs might also be helpful [2].

Surgery is needed if medical therapy is unsuccessful or impractical because of severe illness. Duodenojejunostomy is the most often performed procedure for SMA syndrome [30]. Duodenojejunostomy, which can be performed laparoscopically or via open surgery, is the process of forming a junction between the jejunum and the duodenum to avoid the compression brought on by the SMA [24,30]. Less common surgical treatments for SMA syndrome include division of the ligament of Treitz (Strong's operation), transposition of the SMA, gastrojejunostomy, anterior transposition of the duodenum's third segment, intestinal derotation, and Roux-en-Y duodenojejunostomy [17]. It has been demonstrated that duodenal circular draining, an intricate and invasive open surgical technique first developed and carried out in China, can cause reversed peristalsis to react [31]. SMA syndrome treatment is described in Table 3.

**Table 3:** SMA syndrome treatment. SMA: Superior mesenteric artery; TPN: Total parenteral nutrition.

Feature	Description
Treatment Approach	Medical treatment attempted first, surgery reserved for unsuccessful cases or severe illness
Medical Treatment Goals	Weight gain, resolution of underlying issues, duodenal/gastric decompression
Medical Treatment Methods	Nasogastric tube insertion, Prone or left lateral decubitus positioning, Fluid and electrolyte replacement, Removal of precipitating factors, Nutritional support (TPN), Prokinetic drugs (e.g., Metoclopramide)
Surgery	Necessary if medical treatment fails or unsuitable
Most Common Surgery	Duodenojejunostomy (open or laparoscopic), Creates a bypass between duodenum and jejunum to avoid SMA compression
Less Common Surgeries	Division of ligament of Treitz (Strong's operation), SMA transposition, Gastrojejunostomy, Anterior duodenum transposition, Intestinal derotation, Roux-en-Y duodenojejunostomy
Note	Duodenal circular draining (complex surgery) not recommended due to reversed peristalsis risk

## Conclusions

In conclusion, SMA syndrome presents a diagnostic and therapeutic challenge due to its rare occurrence and diverse clinical manifestations. This review offers a comprehensive overview of the aetiology, symptoms, diagnostic hurdles, and management strategies associated with SMA syndrome. Symptoms can range from early satiety to severe abdominal pain, which often results in malnutrition. Diagnosis requires a thorough gastrointestinal evaluation and various imaging modalities. Management entails both medical interventions, such as nasogastric decompression and nutritional support, and surgical interventions like duodenojejunostomy. Understanding the complexities of SMA syndrome is crucial for timely diagnosis and effective management, especially considering its potential overlap with other gastrointestinal disorders or eating disorders. Further targeted research is warranted to deepen our understanding of this condition and improve patient outcomes.
